# Equipment Identification and Localization Method Based on Improved YOLOv5s Model for Production Line

**DOI:** 10.3390/s222410011

**Published:** 2022-12-19

**Authors:** Ming Yu, Qian Wan, Songling Tian, Yanyan Hou, Yimiao Wang, Jian Zhao

**Affiliations:** 1 School of Computer and Information Engineering, Tianjin Chengjian University, Tianjin 300384, China; 2 School of Control and Mechanical Engineering, Tianjin Chengjian University, Tianjin 300384, China

**Keywords:** YOLOv5s, production line equipment, CA attention module, GSConv, Slim-Neck, Decoupled Head

## Abstract

Intelligent video surveillance based on artificial intelligence, image processing, and other advanced technologies is a hot topic of research in the upcoming era of Industry 5.0. Currently, low recognition accuracy and low location precision of devices in intelligent monitoring remain a problem in production lines. This paper proposes a production line device recognition and localization method based on an improved YOLOv5s model. The proposed method can achieve real-time detection and localization of production line equipment such as robotic arms and AGV carts by introducing CA attention module in YOLOv5s network model architecture, GSConv lightweight convolution method and Slim-Neck method in Neck layer, add Decoupled Head structure to the Detect layer. The experimental results show that the improved method achieves 93.6% Precision, 85.6% recall, and 91.8% mAP@0.5, and the Pascal VOC2007 public dataset test shows that the improved method effectively improves the recognition accuracy. The research results can substantially improve the intelligence level of production lines and provide an important reference for manufacturing industries to realize intelligent and digital transformation.

## 1. Introduction

Since Industry 4.0 was officially in Germany in 2013, the global manufacturing industry development has rapidly progressed towards digitalization and networking. Industry 5.0, proposed in 2021, demands more sophisticated intelligent manufacturing systems and promotes smart factories with human–machine collaboration at its core. Praveen Kumar Reddy Maddikunta et al. [1] discussed potential applications such as smart manufacturing for Industry 5.0. Zhang et al. [2] explored the application of blockchain technology in cloud manufacturing platforms in the era of Industry 5.0. Aros Erick. A et al. [3] stated that developed economies are currently developing deep learning, machine learning, virtual reality, and augmented reality technologies geared towards industry 5.0. Laura et al. [4] found that the technology, manufacturing, and communication industries have already started digital transformation. Jafari et al. [5] used a four-party intelligence framework (i.e., intelligent automation, intelligent devices, intelligent systems, and intelligent materials) for intelligent logistics for Industry 5.0 human–machine collaboration and highlighted the importance of human–machine collaboration, computational methods such as machine learning, deep learning, clustering, and regression. With the digital and intelligent transformation of the global manufacturing industry, the production line as an important part of the manufacturing industry is also undergoing tremendous transformation. In order to control the daily production work in a timely manner, video surveillance technology has been transferred from the field of social security to the intelligent manufacturing arena.

At present, the production line monitoring system can be classified into a traditional monitoring system, sensor, and RFID-based monitoring system. The traditional production line video monitoring is through human monitoring over a long duration and manually recording the distribution, trajectory, and location information of each piece of equipment in the production line. This is not only difficult to ensure the accuracy of equipment information in a large number of complex production environments, but also leads to low work efficiency. In order to resolve the above problems, a sensor- and RFID-based monitoring system was introduced. This kind of monitoring system uses multiple sensors to achieve shop floor monitoring and identification [6,7,8] and uses RFID readers to achieve equipment identification and maintenance [9,10,11,12,13]. However, such monitoring systems usually require a large number of sensors and RFID, and the wiring installation is complicated and prone to high failure rate, high maintenance cost, difficult acquisition, low accuracy rate, and inaccurate position return during production and maintenance.

The recent development in deep learning-based target detection algorithms is increasingly applied in the manufacturing fields, which helps resolve the earlier mentioned limitation. The most commonly used ones include YOLO (You Only Look Once) series, SSD (Single Shot MultiBox Detector) [14] series, R-CNN (Region-Convolutional Neural Networks) [15], and Faster R-CNN [16]. Compared with traditional detection methods, deep learning-based target detection algorithms exhibit high speed, high accuracy, and robustness in complex manufacturing environments. Currently, deep learning-based YOLO series of network models (YOLOv1 [17], v2 [18], v3 [19], v4 [20], v5) are used extensively in the manufacturing industry. For example, Zhang [21] et al. deployed YOLOv2 for auto-identification application in oil industry facilities. Later, Huang [22] et al. introduced YOLOv3 into oil industry facilities and YOLOv3 lightweight improvement was done to achieve fast recognition of electronic components in complex backgrounds. In 2020, Wu [23] proposed an improved YOLOv3 algorithm to detect electrical connector defects. Song [24] et al. later proposed a grasping robot object detection method based on the improved YOLOv5. A robot target detection platform was first designed. Gao [25] et al. applied the YOLOv4 target detection algorithm to a robotic arm grasping system suitable for complex environments. Yan [26] et al. proposed YOLOv3 network for operation area detection in their latest work in 2022. In the same year, Yu [27] et al. used the improved YOLOv3 for defect detection and Luo [28] et al. proposed an engineering diagram recognition method based on YOLOv4 algorithm for the detection of component targets under circuit diagrams. Ge [29] et al. proposed a visual object tracking network based on YOLOv5s, which can be used to detect robots and provide a new idea for target detection in manufacturing. Huang [30] et al. proposed a holistic approach for fractal target detection based on a multi-head model.

The YOLO family of algorithms has also seen applications in the field of intelligent video surveillance. For instance, Yang [31] et al. proposed a target detection and ranging technique based on YOLOv5 and depth cameras and applied it to practical engineering for AGV localization. in 2022, Zou [32] et al. proposed an improved YOLOv5s helmet detection algorithm based on a deep learning approach. This technology was then deployed to implement a set of intelligent monitoring systems for edge terminal maintenance personnel. Very recently, Soma [33] et al. proposed an intelligent video monitoring scheme based on YOLOv3 for detecting people, vehicles, and background objects.

A review of the literature suggests that an accurate/efficient equipment identification and positioning method is highly desirable for production information monitoring, improved productivity, and ensuring orderly production. In response to this need, this paper proposes a production line equipment identification and localization method based on the improved YOLOv5s model, which has been demonstrated for application in a production line energy monitoring system. The proposed method is dedicated to addressing the current problems facing complex manufacturing environments, and will help operators effectively identify the equipment category and position equipment accurately and efficiently in real time.

## 2. Improvement of YOLOv5 Model

### 2.1. The YOLOv5 Model

YOLOv5 is divided into four versions according to the size of the model, namely YOLOv5s, YOLOv5m, YOLOv5l, and YOLOv5x, and the width and depth of each version increases in order. The YOLOv5 model is divided into four layers of structure: Input, Backbone, Neck, and Detect. The input contains three parts: Mosaic data enhancement, adaptive computation of the anchor frame, and adaptive scaling of the image. During training, the input side enriches the dataset by image stitching or overlaying random scaling, in which the interaction ratio is obtained by adaptively computing the anchor frame, and finally the dimensionally consistent image is obtained by adaptive scaling.

The Backbone layer extracts the main feature information. YOLOv5 version 6.0 uses convolutional operations instead of Focus module in previous versions. The backbone layer is mainly divided into Conv module, C3 module, and SPPF module. Conv module in YOLOv5 version 6.0 contains Conv2d, Batch Normalization, and SiLU activation function. C3 module contains 3. The C3 module contains 3 Conv modules and several Bottleneck modules. The Spatial Pyramid Pooling—Fast (SPPF) module replaces the original Spatial Pyramid Pooling (SPP) module in YOLOv5 6.0 and is much faster.

The Neck layer uses Path Aggregation Network (PANet) and Feature Pyramid Networks for feature fusion, fusing features from different layers to detect small and large targets, and is responsible for passing image features to the Detect layer. Among them, Path Aggregation Network (PANet) serves to solve the problem of arbitrary size of input data and increase the perceptual field of the network, and Feature Pyramid Networks improve the detection of small targets.

YOLOv5 makes a prediction for each grid of the feature map, uses the predicted information to compare with the true information, and then decides the next convergence. The loss function is the evaluation criterion for predicted information and the real information, the smaller the loss function is, the closer the predicted information is to the real information. The loss of YOLOv5 mainly contains bbox_loss (Rectangular frame loss), cls_loss (Classification loss), and obj_loss (Confidence loss).

IoU (Interaction Over Union), also known as interaction ratio, is an indication of the degree of overlap between the prediction bounding box and the object bounding box. It is used to determine whether the result has been predicted successfully. A threshold value can be set for IoU, and if the interaction ratio is greater than this threshold, the prediction is considered successful; otherwise, the prediction fails. If the IoU threshold is set too low, it is difficult to guarantee the quality for the detection samples. In order to achieve high quality positive samples, the IoU threshold can be manually adjusted upward, but too high an IoU threshold will lose the small-scale target frame. Therefore, the threshold was generally set to 0.5. IoU is defined in Equation (1).

(1) $$\mathrm{IoU}=\frac{\mathrm{Prediction}\;\mathrm{bounding}\;\mathrm{box}\cap \mathrm{Object}\;\mathrm{bounding}\;\mathrm{box}}{\mathrm{Prediction}\;\mathrm{bounding}\;\mathrm{box}\cup \mathrm{Object}\;\mathrm{bounding}\;\mathrm{box}}$$

When the prediction frame does not intersect with the real frame, the IoU loss is 0, and IoU will affect the model convergence process. As such, YOLOv5 uses CIoU (Complete-IoU) to calculate bbox_loss by default. The loss calculation formula of CIoU is shown in Equation (2).

(2) $$\;L_{\mathrm{CIoU}}=1-\mathrm{IoU}+\frac{\rho^{2}\left(\;{b,b}^{\mathrm{gt}}\right)}{c^{2}}+\alpha v$$

where b and $b^{\mathrm{gt}}$ denote the centroids of the prediction frame and the real frame, respectively, ρ denotes the Euclidean distance between the two centroids, and c denotes the diagonal distance between the minimum closure region of the prediction frame and the real frame. α is the weight parameter as shown in Equation (3). v is used to measure the consistency of the aspect ratio, as shown in Equation (4).

(3) $$\alpha =\frac{v}{(1-\mathrm{IoU})+v}$$

(4) $$v=\frac{4}{\pi^{2}}{\left(\arctan\frac{w^{\mathrm{gt}}}{h^{\mathrm{gt}}}-\arctan\frac{w}{h}\right)}^{2}$$

YOLOv5 uses the binary cross-entropy function to calculate cls_loss and obj_loss by default. The binary cross-entropy function is shown in Equation (5).

(5) $$L=-y\log p-(1-y)\log(1-p)=\{\begin{array}{cc} -\log p & ,y=1\\ -\log(1-p) & ,\;y=0 \end{array}$$

where y is the label corresponding to the input sample (1 for positive samples and 0 for negative samples) and p is the probability that the model predicts this input sample to be positive.

**Figure 1:**
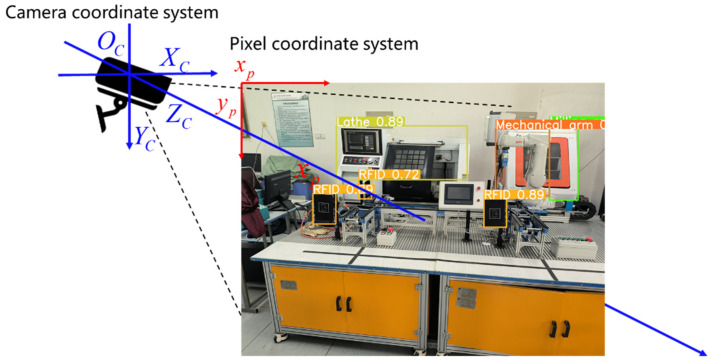
The pixel coordinate system and the camera coordinate system.

The YOLOv5 model is able to output the position information of the detected target while recognizing the target. The color image and depth image are generated when using RGB-D camera for detection, and the position coordinates are obtained by converting the pixel coordinate system to the camera coordinate system to obtain the 3D position coordinates of the detection target. In order to obtain more reliable depth information, the RGB-D camera is usually calibrated for depth before detection [34,35]. The pixel coordinate system and the camera coordinate system are shown in Figure 1. Usually, the upper-left pixel point of the image is used as the origin position of the pixel coordinate system, and the pixel coordinate system axes are shown as the red arrows in Figure 1. The center coordinate point of the detected target is obtained and projected into the pixel coordinate system of the depth image, so the pixel value of the center coordinate point of the detected target in the depth image is the distance of the detected target to the RGB-D camera. Taking the RGB-D camera as the origin of the camera coordinate system, the camera coordinate system axes are shown as the blue arrows in Figure 1.

YOLOv5 has been widely used in the field of pedestrian detection and has achieved good results. For the detection of production line equipment that changes dynamically in real time in a complex background, we cite YOLOv5, which has a more concise network structure and faster processing speed, as a baseline network model for production line equipment identification and localization improvement.

### 2.2. Improvement Strategies for the YOLOv5 Model

#### 2.2.1. Adding CA (Coordinate Attention) Attention Module

A survey of the literature shows that soon after the emergence of attention mechanisms, attention mechanisms such as SE (Squeeze-and-Excitation) [36], CBAM [37], and CA (Coordinate Attention) [38] have been widely applied to the field of deep learning. Cui [39] et al. proposed a method based on Gramian Angular Field (GAF) and CA-based lightweight rolling bearing fault diagnosis method to reduce the computational effort and validate the effectiveness of adding CA attention module to neural networks. Zhang [40] et al. proposed CaNet, a deep learning network for identifying concrete cracks, which added a CA attention module to a model with ResNet50 as the backbone network. The result sees a significant improvement in recall, F1 score, and accuracy, which experimentally verified that the addition of CA attention module can effectively improve the system accuracy. Cheng [41] et al. proposed a lightweight crop pest detection method based on convolutional neural networks, using YOLOLite as the backbone network and lightweight hourglass blocks and CA attention module to optimize the structure of the residual blocks, and the precision was greatly improved. Wang [42] et al. proposed the CA-EfficientNetV2 model, adding the CA attention module to the head of the EfficientNetV2 model to enhance the classification effect and thus enabling efficient feature learning. Several experimental studies demonstrated the effectiveness of CA attention module to the neural network framework for precision improvement.

The CA attention module not only captures the exact position of the object of interest when learning features and constructing channel attention, but also has the same features as attention mechanisms.

**Figure 2:**
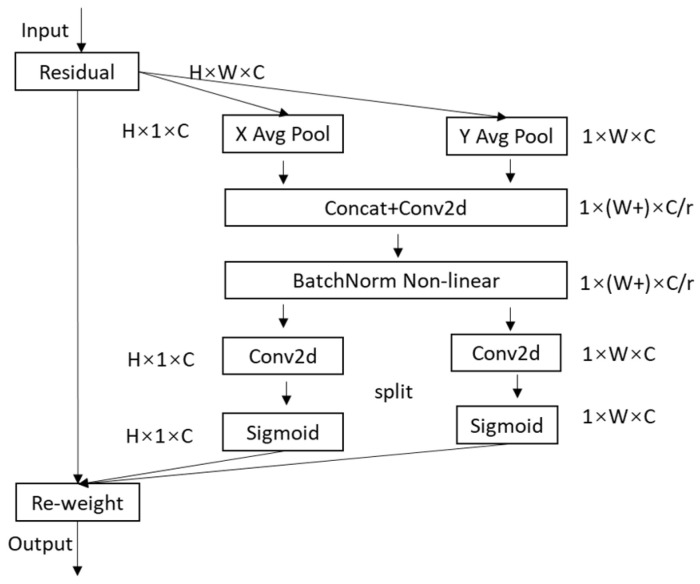
Implementation process of CA attention module.

The primary role of the CA attention module is to enhance the expression of the learning features of the mobile network. Any intermediate feature tensor in the model is input and then transformed to an output tensor of the same size, as shown in Equation (6). The implementation process of the CA attention module is shown in Figure 2, C represents the input image’s channel value, H is the length of the input image, W is the width of the input image, and r is the scaling ratio.

(6) $$X=[x_{1},x_{2},\ldots ,x_{c}]\in R^{H\times W\times C}$$

The CA attention module obtains the feature maps in both width and height directions by performing global average pooling in both directions after inputting the feature maps, The output of the cth channel with input x, height h, and width w is shown in Equations (7) and (8).

(7) $$z_{c}^{h}(h)=\frac{1}{W}\sum_{0\leq i\leq W}|x_{c}(h,j)$$

(8) $$z_{c}^{w}(w)=\frac{1}{H}\sum_{0\leq j\leq H}|x_{c}(j,w)$$

After that, the channel merging operation along the spatial dimension was performed on the feature map, and then it was transformed using the convolutional transform function as shown in Equation (9), where δ is the nonlinear activation function and f is the intermediate feature map used to encode the spatial information in the horizontal and vertical directions.

(9) $$f=\delta (F1([z^{h},z^{w}]))$$

The decomposition of *f* into two separate tensors *g^h^* and *g^w^*, along the spatial dimension, is shown in Equations (10) and (11), where σ is the sigmoid activation function usually used to reduce the number of channels of *f* by scaling down r to reduce the model complexity.

(10) $$g^{h}=\sigma (F_{h}(f^{h}))$$

(11) $$g^{w}=\sigma (F_{w}(f^{w}))$$

Following Equations (10) and (11), input feature map of the attention weights in the height direction (*g^h^*) and the attention weights in the width direction (*g^w^*) can be obtained. Finally, the output feature map $y_{c}(i,j)$ in the CA is shown in Equation (12).

(12) $$y_{c}(i,j)=x_{c}(i,j)\times g_{c}^{h}(i)\times g_{c}^{w}(j)$$

#### 2.2.2. Introducing GSConv and Slim-Neck Methods in the Neck Layer

The current target detection model cannot fully meet the requirements of real-time detection of intelligent monitoring systems with high precision. We added GSConv and Slim-Neck methods to the YOLOv5 network model to improve the precision and other indicators of the model. Similar methods were proposed by Hulin Li [43] et al. in the application of self-driving cars.

**Figure 3:**
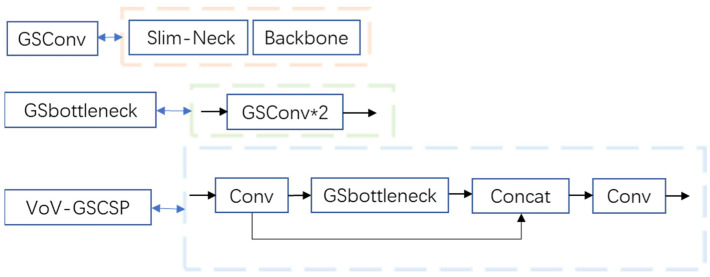
Structure of GSConv and VoV-GSCSP modules.

GSConv and Slim-Neck methods were added to the YOLOv5-6.0 network model by replacing Conv in the Neck layer of the YOLOv5-6.0 version of the network with the lightweight convolutional method GSConv. YOLOv5 backbone feature extraction network adopts a C3 structure, with many parameters in the training process. The use scenarios are easily restricted in the use scenarios such as intelligent monitoring systems. With challenges in applying intelligent surveillance systems in mobiles and embedded usage settings, the C3 module is replaced by the VoV-GSCSP module. The structure of GSConv and VoV-GSCSP modules is shown in Figure 3.

#### 2.2.3. Detect Layer Adds Decoupled Head Structure

The inherent conflict between identifying the target class (classification problem) and determining the target location (regression problem), which are the two main tasks in target detection, also limits the model performance to some extent. The YOLO series networks use coupled detection heads in the prediction part to accomplish the tasks of identifying the target class and determining the target location simultaneously. The task of identifying the target category is concerned with which existing category the texture features of the target are most similar to and determining the target location is concerned with the edge features of the target for bounding box parameter correction. The different objectives of the two tasks lead to different solutions, so the choice of the detection head has a certain impact on the performance of the model.

**Figure 4:**
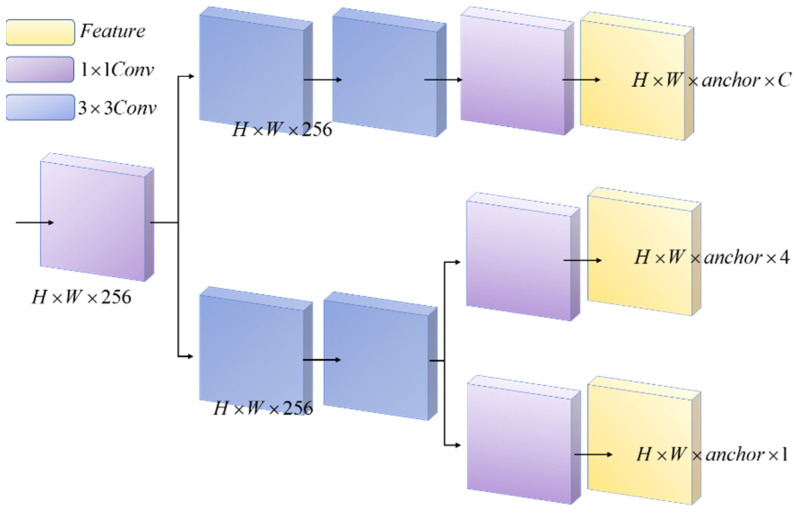
Structure of Decoupled Head.

Song et al. [44] investigated the inherent conflict between target detection classification and position regression. Zheng et al. [45] improved YOLOV3 by adding Decoupled Head, which improved the performance metrics of the model, and found that the coupled detection head of the YOLO series network degraded the performance to some extent. Li [46] et al. improved YOLOv4 by decoupling the classification and regression tasks to enhance the performance of the model and applied to the detection of ship targets. The structure of the Decoupled Head is shown in Figure 4. The Decoupled Head goes through a 1 × 1 convolution operation and then two parallel 3 × 3 convolution operations, one of which passes through a 1 × 1 convolution layer and is dimensionally reduced to complete the task of identifying the target class, and the other passes through two 3 × 3 convolution layers and then uses two parallel 1 × 1 convolutions for the task of target location and confidence. In summary, Decoupled Heads improves the performance of the model by solving the target category (classification problem) and determining the target location (regression problem) separately.

We use the Decoupled Head in the Detect layer of the YOLOv5 model. We decoupled the coupled detection heads in the prediction part of the original model to perform the target detection classification task and the position regression task separately.

### 2.3. A Framework of Production Line Equipment Identification and Localization Method Based on Improved YOLOv5s Model

In order to achieve accurate recognition and classification, this paper proposes a production line equipment recognition and localization method based on an improved YOLOv5s model. Aiming at the problems that the model is not accurate enough for object localization and the weak expression ability of model learning features, the CA attention module was introduced into the YOLOv5s network model architecture. To address the problems of easy overfitting, slow training speed, and excessive parameters in the training process, we replaced Conv in the Neck layer with the lightweight convolution method GSConv and introduced the Slim-Neck method. To improve the performance index of the model for the inspection of production line equipment, we used the Decoupled Head in the Detect layer of the YOLOv5 model.

**Figure 5:**
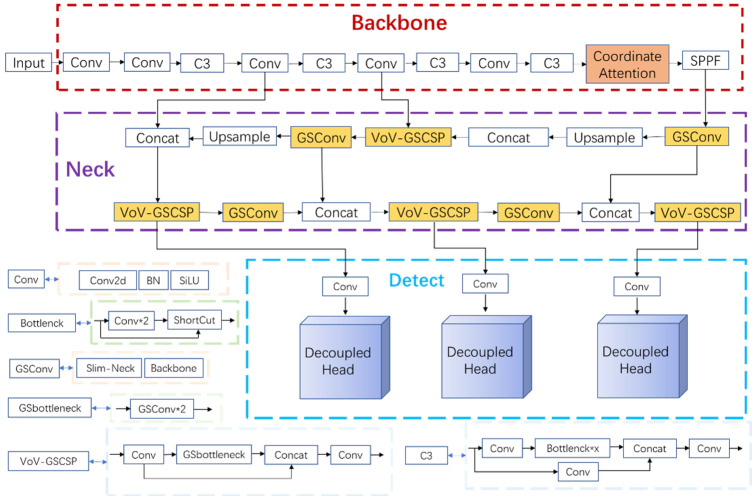
Network structure of production line equipment identification and localization method based on improved YOLOv5s model.

The model of this method consists of Input, Backbone, Neck, and Detect. The Backbone layer mainly performs feature extraction, which extracts the object information from the image through the convolutional network for later target detection. The Neck layer blends and combines the features to enhance the robustness of the network and strengthen the object detection ability and passes these features to the Head layer for prediction. To improve training speed, YOLOv5s version 6.0 replaces the Focus module with a convolution operation of size 6 × 6, step size 2, and padding 2. The Bottleneck module is based on the residual structure of ResNet, which can effectively reduce the training time. The C3 module contains three standard convolutional layers and multiple Bottleneck modules. This way, remote dependencies can be captured along one spatial direction, while accurate location information can be preserved along the other spatial direction. The generated feature maps are then encoded as a pair of orientation-aware and position-sensitive attention maps, respectively, which can be applied complementarily to the input feature maps to enhance the representation of objects of interest. Version 6.0 of YOLOv5s uses the SPPF module instead of the SPP (Spatial Pyramid Pooling) module. The SPPF module uses multiple small-sized pooling kernels in cascade instead of a single large-sized pooling kernel in the SPP module. GSConv and Slim-Neck methods are introduced in Neck layer. On the one hand, it replaces the Conv module with the lightweight convolution method GSConv, and on the other hand, it replaces the previous C3 module with the VOV-GSCSP module, which consists of the GSbottleneck module and Conv module, which are set up by GSConv. Finally, the three higher resolution features from the fused features are input to the decoupling head to complete the task of identifying the target class (classification problem), determining the target location (regression problem) and the confidence level. The improved network model and structure are shown in Figure 5.

## 3. Production Line Equipment Identification Experiment

### 3.1. Build the Experimental Platform

A highly configured deep learning server benefits from high performance and improved precision rate. Therefore, NVIDIA RTX A5000 was chosen as the computing GPU for the experiments. Table 1 shows the representative environment versions required for the experiments.

### 3.2. Making the ProductionIineData Dataset

The datasets were obtained from the simulated production line in the Intelligent Manufacturing Technology Laboratory of the School of Control and Mechanical Engineering of Tianjin Chengjian University. Six devices (AGV smart cart, AGV Raspberry Pie cart, mechanical arm, RFID, lathe, and milling machine) of the simulated production line were selected for real-time inspection in the experiment. The datasets used for the experiments are (i) field dataset collection, (ii) dataset labeling, and (iii) dataset construction.

(i) Field dataset collection: In order for the model to learn more features of the six devices, the dataset images were taken by CCD cameras at various angles, under different environmental conditions, at different times, and in various backgrounds and cropped to multiple sizes (1706 × 1280 px, 2844 × 1280 px, 1280 × 1706 px, 421 × 391 px). After collation, 500 photographs of each size shot under varied situations were acquired, and 50 background pictures without the production line components were generated.

(ii) Dataset labeling: The dataset labeling is the prerequisite for the algorithmic model to complete supervised learning. This requires manually labeling the object locations of interest and tagging them with categories in the training and validation dataset images. The labeled images are constructed and input into the model to obtain the model weights, and the algorithmic model can recognize the object categories after loading the corresponding model weights.

**Figure 6:**
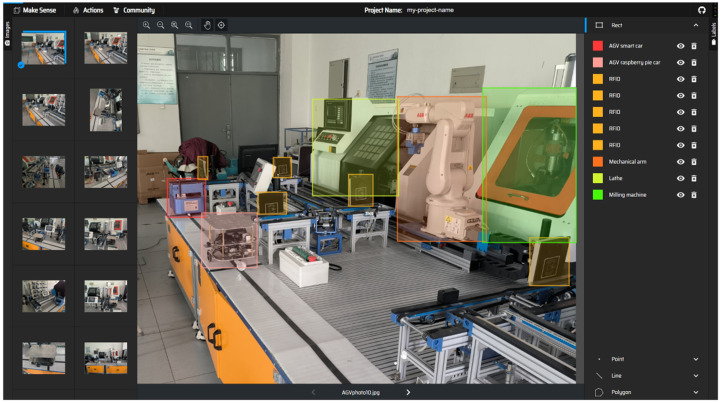
The dataset labeling process.

**Figure 7:**
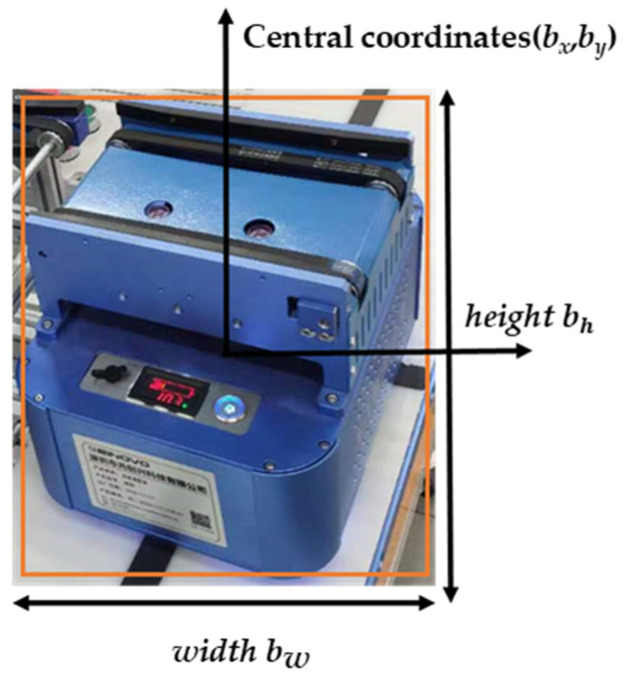
Parameters generated during dataset labeling.

Make Sense was selected as the image labeling tool for this dataset, see Figure 6, before the final labeling information was exported. The labeling information includes the position coordinates of the object of interest in the image and the category information. As shown in Figure 7, object information contains the center position coordinates $\left(b_{x},b_{y}\right)$, height $\left(b_{h}\right)$, width $\left(b_{w}\right)$, and whether the object contains a target and the Confidence of the contained target in the information of each object.

The formula for Confidence is shown in Equation (13).

(13) Confidence=X∗IoU

X indicates whether the target is included or not; if it is included, X = 1; otherwise, X = 0. The formula for calculating IoU is shown in Equation (11).

(iii) Dataset construction: Based on the cross-validation method, 70% of all images are used as the training set and 30% as the validation set. This dataset divides all images into ten copies with 55 images in each copy. The training set contains 385 images and the validation set contains 165 images. The training set was then used to train the algorithm model under various improvement methods to obtain the model weights for different recognition effects.

### 3.3. Evaluation of the Model’s Performance Indicators

**Figure 8:**
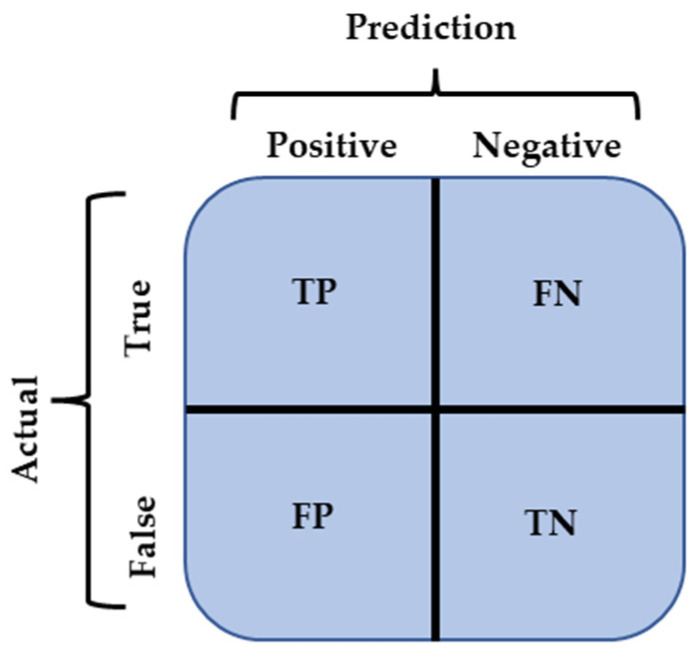
Model metrics judgment.

In the field of target detection, the performance of algorithmic models is usually judged by Precision, Recall, F1 score, IoU (Intersection Over Union), P–R curve (Precision–Recall curve), AP (Average Precision), and mAP (Mean Average Precision). These indicators are calculated from Precision and Actual, and the model indicators are judged as shown in Figure 8. Figure 8 corresponds to four cases: (1) TP indicates that the true category of the sample is positive and the model predicts a positive result, then the result is predicted correctly; (ii) TN indicates that the true category of the sample is negative and the model predicts a negative result, then the result is predicted correctly; (iii) eFP indicates that the true category of the sample is negative, but the model predicts a positive result, then the result is predicted incorrectly; (iv) FN indicates that the true category of the sample is positive and the model where FN indicates that when the true category of the sample is positive and the model predicts a negative outcome, the outcome is incorrectly predicted.

Precision represents the ratio of correct samples predicted to correct samples to all correct samples, as shown in Equation (14).

(14) $$\mathrm{Precision}=\frac{\mathrm{TP}}{\mathrm{TP}+\mathrm{FP}}$$

Recall represents the ratio of the number of correct samples predicted to be correct to the number of all correct samples, as shown in Equation (15).

(15) $$\mathrm{Recall}=\frac{\mathrm{TP}}{\mathrm{TP}+\mathrm{FN}}$$

The P–R curve (Precision–Recall curve) is a graph with Recall as the x-axis and Precision as the y-axis, in which one can find the points where both precision and recall approach 1. The P–R curve graph is also an important indicator to judge the performance of the algorithm model.

AP (Average Precision) is also a mainstream metric to judge the goodness of an algorithm model, which can be approximated as the integration area under the P–R curve. Therefore, the larger the AP value, the more the precision and recall in the P–R curve converge to 1, and the better the algorithm model, as shown in Equation (16).

(16) AP=∫(P−R curve)

mAP (Mean Average Precision) is a combined judgment of the performance of the algorithm model by averaging the APs of several categories of the prediction together.

## 4. Experimental Results of Identification of Production Line Equipment

In this experiment, Precision, Recall, mAP@0.5 (the average precision rate when the IoU threshold is 0.5), and mAP@0.5:0.95 (the average precision rate when the IoU threshold is 0.05 from 0.5 to 0.95 in steps of 0.05) were used as the metrics to evaluate the model. The scene application of the recognition class has higher requirements for Precision, so this experiment used Precision as the first evaluation index.

### 4.1. Experimental Analysis of Equipment Identification in the Production Line

In the experiments, the performance baseline was first obtained under the default configuration of YOLOv5-6.0, (default epochs = 300, batch_size = 16, and img-size = 640. The default configuration of the optimizer is SGD. The algorithm model training results were viewed using Tensorboard, and the model performance baseline results are shown in Table 2.

In order to enhance the model’s accurate localization of the object of interest and to enhance the model’s ability to learn the expression of features, CA (Coordinate attention) attention mechanism, SE attention mechanism (Squeeze-and-Excitation Networks), CBAM (Convolutional Block Attention Module), and ECA (Efficient Channel Attention) were added to the Backbone layer of the model. Table 3 shows the performance comparison after adding the four attention modules. Experimental results show that the model with the addition of the CA attention module achieves 92.8% for accuracy, 83.2% for recall, 88.6% for mAP@0.5, and 59.2% for mAP@0.5:0.95, meaning that a better overall performance was achieved.

In order to further improve the model precision and enhance the expression of the learned features of the model, CA (Coordinate attention) attention mechanism was added to the Backbone layer of the model and the CA (Coordinate attention) attention mechanism, SE attention mechanism (SE) to the C3 module of the Neck layer (Squeeze-and-Excitation Networks), CBAM (Convolutional Block Attention Module), and ECA (Efficient Channel Attention) were added and the results were compared in terms of parameters. Table 4 shows the performance comparison after adding the four attention modules. Results show that the performance of the model with the addition of the four attention modules is lower compared to that before the addition.

To meet the requirements of real-time detection of intelligent monitoring systems and reduce the number of parameters in the training process, we introduced the lightweight convolution method GSConv and Slim-Neck method in the Neck layer. The Conv in the Neck layer of the YOLOv5-6.0 version of the network with the lightweight convolution method GSConv was replaced, as well as the C3 module with the VoV-GSCSP module. Table 5 shows the performance comparison before and after the model modification. Experimental verifications suggest that the introduction of the lightweight convolution method GSConv and Slim-Neck method based on adding CA (Coordinate attention) attention mechanism to the Backbone layer of the model, replacing Conv in the Neck layer of the YOLOv5-6.0 version of the network with the lightweight convolution method GSConv, and replacing the C3 module with the VoV-GSCSP module can further improve the detection performance of the model substantially. After introducing the lightweight convolutional method GSConv and Slim-Neck method in the Neck layer, the model achieves 94.5% for precision and 82.8% for recall, mAP@0.5 achieves 88.5%, and mAP@0.5:0.95 achieves 59.3%, and the precision and recall are greatly improved compared with the original YOLOv5-6.0 model.

To further improve the performance metrics and convergence speed of the model, we added the Decoupled Head structure to the Detect layer of the model. The coupled detection head in the Decoupled Detection layer of the Neck layer of the YOLOv5-6.0 version of the network was decoupled to perform the target detection classification task and the position regression task, respectively. Table 6 shows the performance comparison between the improved model and before the improvement. The accuracy rate of the model after adding the Decoupled Head structure reaches 93.6%, the recall rate reaches 85.6%, and mAP@0.5 reaches 91.8%. On the basis of maintaining the accuracy rate, the recall rate was again improved by 2.8% and mAP@0.5 was again improved by 3.3%. Compared with the original YOLOv5-6.0 model, the improved model had a 4.9% improvement in accuracy, 4.9% improvement in recall, and 3.3% improvement in Map@0.5, resulting in a significant improvement in performance.

### 4.2. Performance Comparison

In order to verify the performance of the production line equipment identification and localization method based on the improved YOLOv5s model, we used the productionlineData homemade dataset performance comparison, Pascal VOC2007 public dataset performance comparison, and recognition test comparison to compare and validate the results, respectively.

#### 4.2.1. Performance Comparison Using ProductionlineData Homemade Dataset

Firstly, the experimental data using the same dataset for YOLOv3, YOLOv5-6.0, YOLOv5-5.0, YOLOv5-Lite, and the model trained based on the improved method were compared, see data in Table 7.

**Figure 9:**
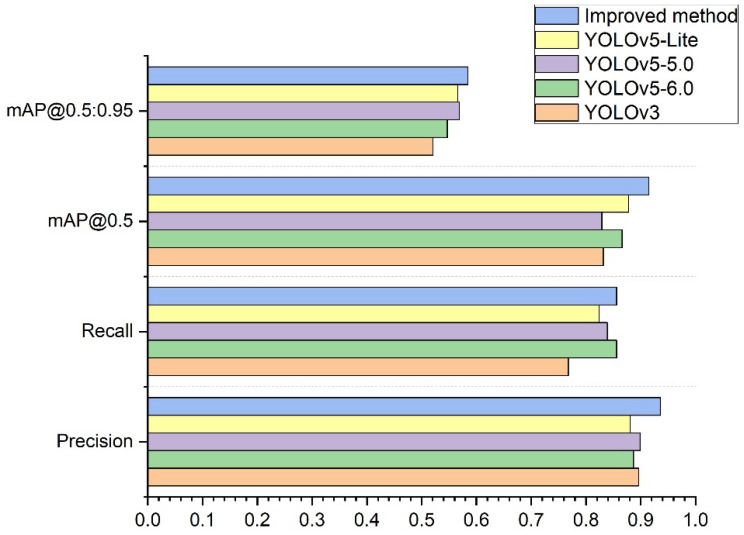
Comparison of performance parameters of YOLOv3, YOLOv5-6.0, YOLOv5-5.0, YOLOv5-Lite, and improved method iteration process.

**Figure 10:**
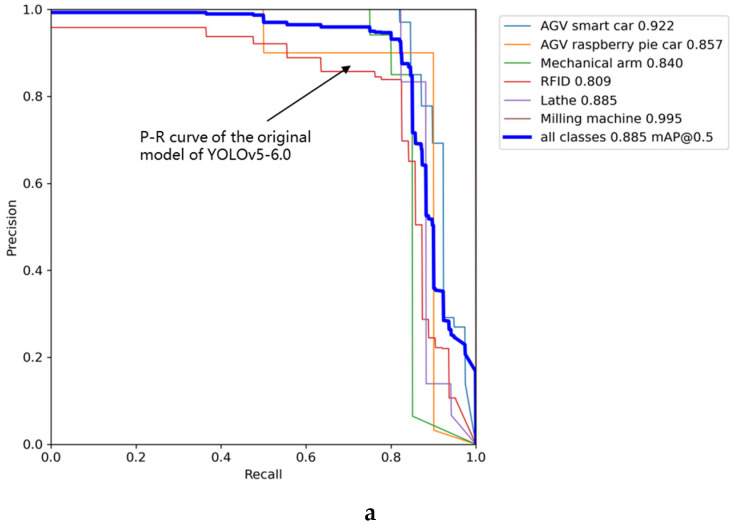

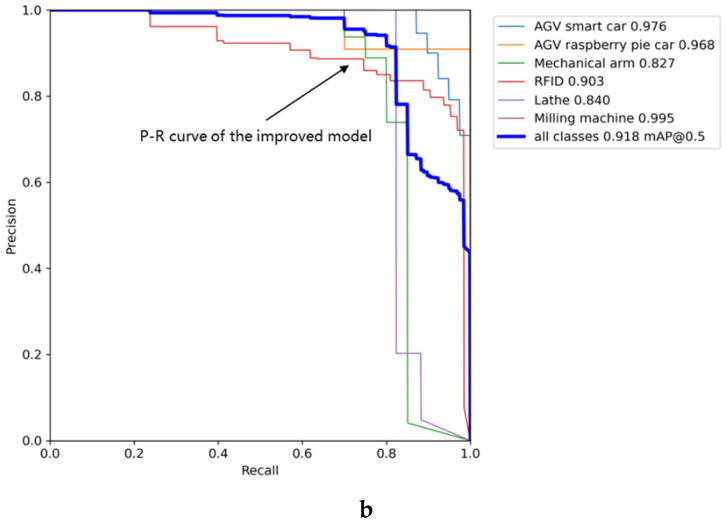
Comparison of the P–R curve of the improved model and the original model of YOLOv5-6.0.

Table 7 shows that when the model trained using the improved method is tested, the precision rate reaches 93.6%, the recall rate reaches 85.6%, mAP@0.5 reaches 91.8%, and mAP@0.5:0.95 reaches 58.5%. The precision rate improved by 5.5% compared to YOLOv5-Lite, the recall rate improved by 8.8% compared to YOLOv3, mAP@0.5 improved by 8.9% compared to YOLOv5-5.0, and mAP@0.5:0.95 improved by 6.4% compared to YOLOv3. The size of the weight of the model trained by the improved method is only 23% of the YOLOv3 model. Figure 9 compares the performance parameters of the iterative process of YOLOv3, YOLOv5-6.0, YOLOv5-5.0, YOLOv5-Lite, and the improved method. It can be seen that the improved method achieved a substantially higher precision rate as well as higher recall rate and other indicators. A comparison of the P–R curve of the improved model with the original YOLOv5-6.0 model is shown in Figure 10. Figure 10a represents the P–R curve of the original model of YOLOv5-6.0, and Figure 10b represents the P–R curve of the improved model. In Figure 10 we can see that the improved model outperforms the original model of YOLOv5-6.0 in terms of mAP@0.5.

**Figure 11:**
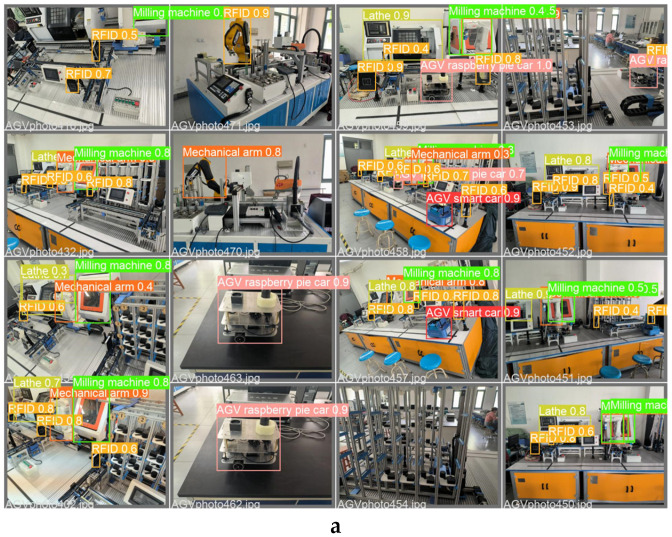

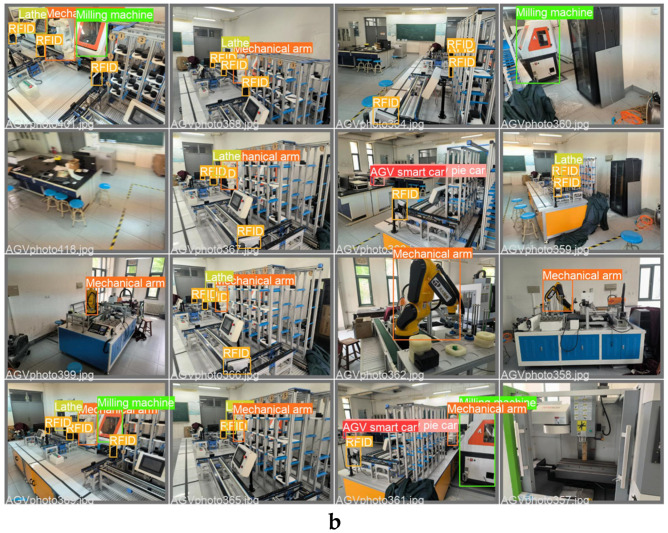
Representative images showing the training process of the improved method.

YOLOv3, YOLOv5-6.0, YOLOv5-5.0, YOLOv5-Lite, and the improved method using productionlineData homemade dataset test results show that the improved method is overall better than YOLOv3, YOLOv5-6.0, YOLOv5-5.0, and YOLOv5-Lite The trained results and the weights obtained by the improved method take up less memory and are more convenient for use with development boards in the industry. Figure 11a,b show representative images taken during the training process. In Figure 11a, it can be seen that the confidence of the improved method is close to 1 for all the devices in the production line recognition. Figure 11b shows that in the improved production line, all equipment have been recognized correctly under the tiny recognition target and complex environment, and the target detection envelope can tightly surround the recognition target.

#### 4.2.2. Performance Comparison Using Pascal VOC2007 Public Dataset

The standard dataset of VOC2007 from The PASCAL Visual Object Classes is a benchmark to measure the performance of image classification recognition. The dataset contains training set travel (5011 images), test set test (4952 images), a total of 9963 images, containing 20 categories such as airplane, bicycle, bird, and boat.

**Figure 12:**
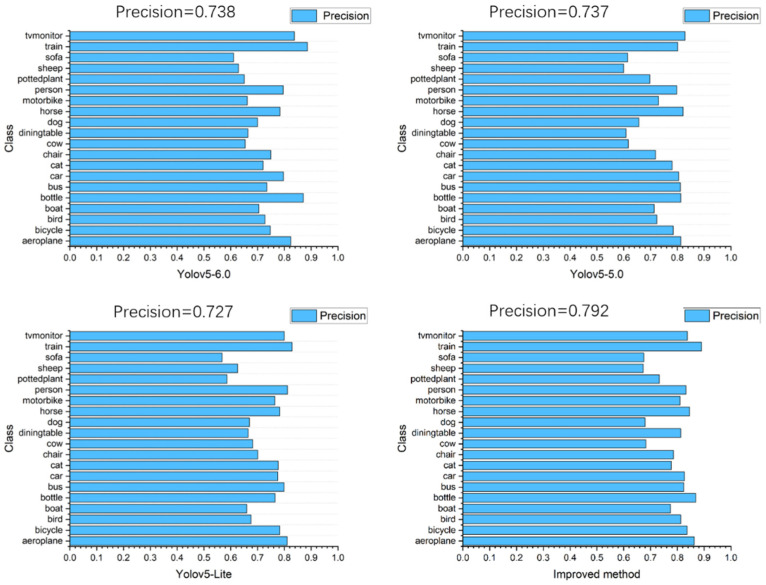
Precision rate of YOLOv5-6.0, YOLOv5-5.0, YOLOv5-Lite, and improved method model weights.

In this paper, we used Pascal VOC2007 public dataset to compare the experimental data of the models trained by YOLOv5-6.0, YOLOv5-5.0, YOLOv5-Lite, and the improved method, and the results are shown in Table 8. For Pascal VOC2007 public dataset trained using the improved method, the precision rate reaches 79.2%, the recall rate reaches 59.5%, mAP@0.5 reaches 66.8%, and mAP@0.5:0.95 reaches 44.1%. Compared with YOLOv5-Lite, the precision rate improved by 6.5%, and compared with YOLOv5-5.0, mAP@0.5 improved by 1.5%. Figure 12 shows the precision rates of the model weights for YOLOv5-6.0, YOLOv5-5.0, YOLOv5-Lite, and the improved method.

Comparing the test results of YOLOv5-6.0, YOLOv5-5.0, YOLOv5-Lite, and the improved method using the Pascal VOC2007 public dataset show that the improved method generally outperforms the results trained by YOLOv5-6.0, YOLOv5-5.0, and YOLOv5-Lite. The improved method is faster to train, has fewer parameters and higher accuracy, and is more convenient to use with development boards in industrial target detection.

#### 4.2.3. Comparison of Simulated Production Line Scene Recognition Test Results

**Figure 13:**
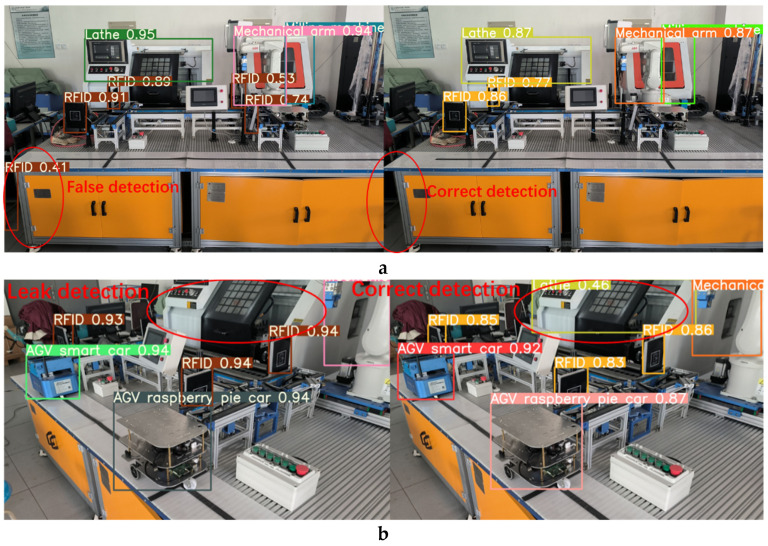
Comparison of recognition test results between YOLOv5-5.0 and the improved method.

**Figure 14:**
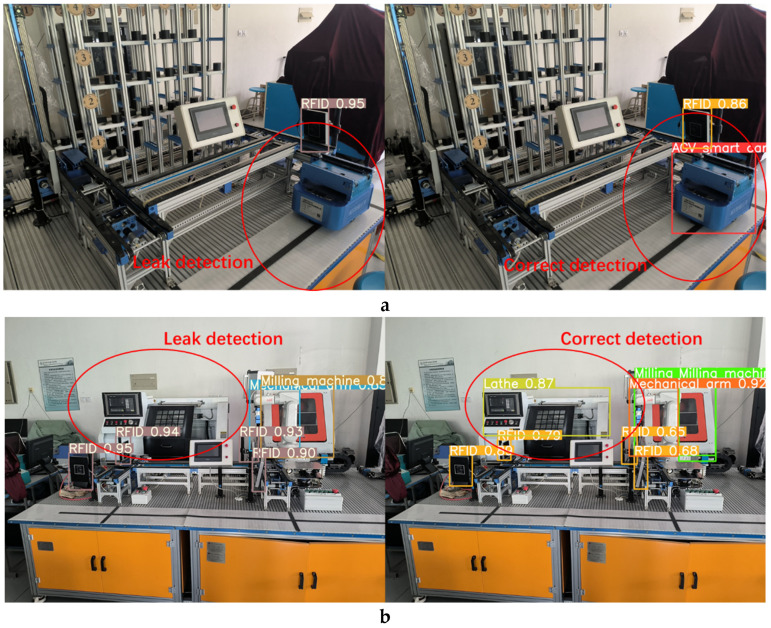
Comparison of recognition test results between YOLOv5-6.0 and the improved method.

**Figure 15:**
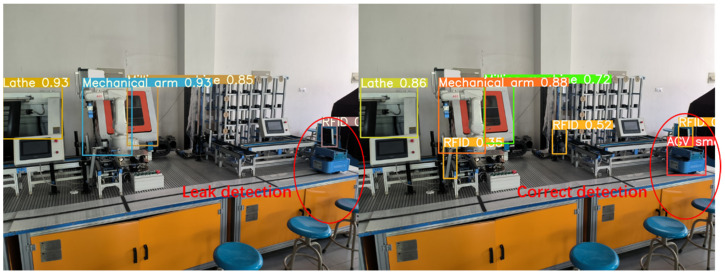
Comparison of recognition test results of YOLOv3 and the improved method.

**Figure 16:**
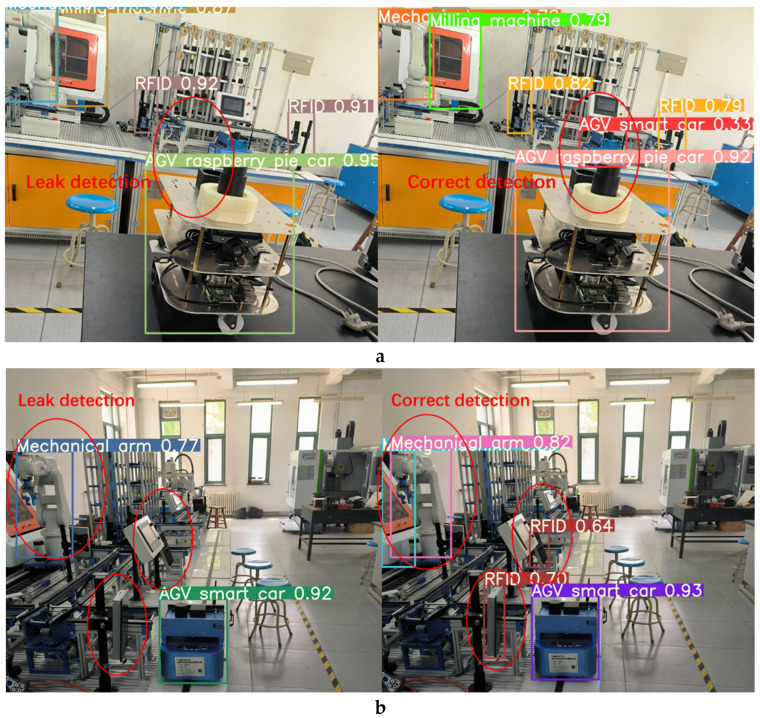
Comparison of recognition test results between YOLOv5-Lite and the improved method.

In order to verify the performance of the production line equipment identification and localization method based on the improved YOLOv5s model, we introduced weights trained by YOLOv3, YOLOv5-6.0, YOLOv5-5.0, YOLOv5-Lite, and the improved method into identification tests, and the comparison of the test results are shown in Figure 13, Figure 14, Figure 15 and Figure 16. In Figure 13a, it can be seen that the YOLOv5-5.0 model incorrectly identifies the black shadow part in the lower left corner as RFID, and there is a false detection. In Figure 13b, the YOLOv5-Lite model weights do not identify the lathe, and there is a missed detection. In contrast, the improved method identifies all parts correctly without missing detection. Figure 14a shows that the YOLOv5-6.0 model weights do not identify the AGV smart car. In Figure 14b, the YOLOv5-6.0 model weights do not identify the lathes, and there is a leakage situation. In contrast, the improved method identifies all parts correctly without leakage. A reliable 3D coordinate positions of the equipment is also obtained through the conversion from pixel coordinate system to camera coordinate system. The model weights of YOLOv3 and YOLOv5-Lite in Figure 15 and Figure 16a do not identify the AGV smart car. The model weights of YOLOv5-Lite in Figure 16b do not identify the milling machines and RFID. In contrast, the improved method identifies all correctly without missing detection. For all cases, the center coordinates of all equipment can be accurately transmitted back to the console by the improved method, showing its more reliable performance.

**Figure 17:**
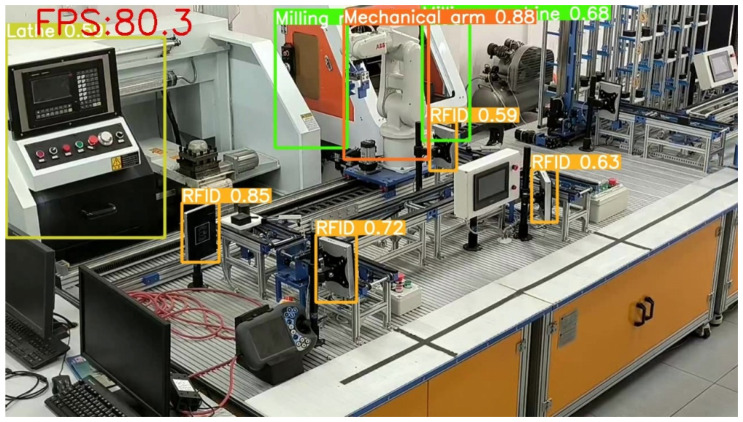
FPS test results of the improved method.

When using sensors and RFID to monitor production line equipment, firstly, a large number of sensors need to be arranged, and secondly, data integration is required during the inspection process, and it is difficult to achieve real-time detection. Traditional manual monitoring of production line equipment is inefficient, inaccurate, and fails to ensure staff safety. The working environment using the improved method can intelligently identify the production line equipment based on the location and category information provided by the monitoring system, expanding the flexibility of the manufacturing process. It greatly reduces the cost of manpower and equipment, effectively avoids the subjectivity and individual differences in the manual inspection process, and provides higher inspection efficiency and accuracy. Experiments show that the FPS (Frames Per Second) of the improved method can reach 80.3, meeting the requirements of real-time inspection, as shown in Figure 17.

## 5. Conclusions

This paper proposes an improved YOLOv5s model-based production line equipment identification and localization method for production line equipment identification and localization. The method effectively improves the precision rate and other production line equipment identification indexes by introducing the CA attention module in YOLOv5s network model architecture, the lightweight convolutional GSConv and the Slim-Neck method in the Neck layer, and adding Decoupled Head structure to the Detect layer. Using productionlineData homemade dataset test, the results show that the precision rate of the improved method reaches 93.6%. The recall rate reached 85.6%, mAP@0.5 reached 91.8%, and mAP@0.5:0.95 reached 58.5%. Compared with YOLOv5-Lite, the precision rate is improved by 5.5%. The recall rate increased by 8.8% compared to YOLOv3. 8.9% improvement in mAP@0.5 compared to YOLOv5-5.0. 6.4% improvement in mAP@0.5:0.95 compared to YOLOv3. The improved method achieves 79.2% precision and 59.5% recall in Pascal VOC2007 public dataset test results, mAP@0.5 reaches 66.8%, and mAP@0.5:0.95 reaches 44.1%. Compared to YOLOv5-Lite, the precision rate improved by 6.5%. Compared to YOLOv5-5.0, mAP@0.5 improved by 1.5%, and mAP@0.5:0.95 improved by 2.1%. During the model test, the method could accurately identify and return the center coordinate positions of all production line devices and obtain the 3D coordinate positions of the devices by converting the pixel coordinate system to the camera coordinate system. The method has high real-time and recognition accuracy, with smaller model weight, and hence is more suitable for industrial production scenarios. The authors acknowledge the potential limitations in the current model. For instance, some frame screens with small equipment target missed detection. The complex production line environment could easily disturb the identification process. Future work will be dedicated to further improving the recognition and detection capability of the model.

This work provides a potential solution towards low cost, low maintenance, and high precision production line monitoring system for complex manufacturing environments. It can enhance the intelligence and automation in the manufacturing industry such as multi-equipment production lines and can potentially transform the traditional manufacturing industry into intelligent manufacturing. The method proposed here can also be generalized to other production scenarios, such as the identification and localization of products in the production process, and the localization identification and obstacle recognition of transportation equipment inside production lines such as AGVs [47] and AMRs [48] (Autonomous Mobile Robot) in the production process.

## Figures and Tables

**Table 1:** Partial environment configuration table required for the experiment.

Name of Development Environment	Configuration Versions
Ubuntu	20.04
Cuda	11.3
python	3.8
Numpy	1.21.6
Opencv	4.1.2
PyTorch	1.10.0

**Table 2:** Dataset test results for the performance baseline.

Precision	Recall	mAP_0.5	mAP_0.5:0.95
0.887	0.807	0.885	0.607

**Table 3:** Comparison of the models with CA, SE, CBAM, and ECA attention modules added to the Backbone layer.

Attention Mechanism	Precision	Recall	mAP@0.5	mAP@0.5:0.95
Add CA	0.928	0.832	0.886	0.592
Add SE	0.923	0.828	0.883	0.578
Add CBAM	0.915	0.837	0.873	0.582
Add ECA	0.868	0.798	0.862	0.525

**Table 4:** Comparison of the models with the four attention modules CA, SE, CBAM, and ECA added to the C3 module of the Neck layer.

Attention Mechanism	Precision	Recall	mAP@0.5	mAP@0.5:0.95
Add CA	0.868	0.811	0.872	0.543
Add SE	0.921	0.824	0.816	0.571
Add CBAM	0.886	0.795	0.83	0.537
Add ECA	0.884	0.843	0.878	0.541

**Table 5:** Comparison of the models after the introduction of GSConv and Slim-Neck methods in the Neck layer with the model before the improvement.

Model	Precision	Recall	mAP@0.5	mAP@0.5:0.95
YOLOv5-6.0	0.887	0.807	0.885	0.607
Add CA	0.928	0.832	0.886	0.592
Add CA + GSCONV + Slim Neck	0.945	0.828	0.885	0.593

**Table 6:** Comparison between the improved model after adding Decoupled Head structure to Detect layer and the improved model before.

Model	Precision	Recall	mAP@0.5	mAP@0.5:0.95
YOLOv5-6.0	0.887	0.807	0.885	0.607
Add CA	0.928	0.832	0.886	0.592
Add CA + GSCONV + Slim Neck	0.945	0.828	0.885	0.593
Decoupled Head + CA + GSCONV + Slim Neck	0.936	0.856	0.918	0.585

**Table 7:** Comparison of test results of YOLOv3, YOLOv5-6.0, YOLOv5-5.0, YOLOv5-Lite, and the improved method using the productionlineData homemade dataset.

Model	Weights	Precision	Recall	mAP@0.5	mAP@0.5:0.95
YOLOv3	117 MB	0.896	0.768	0.832	0.521
YOLOv5-6.0	13.7 MB	0.887	0.807	0.885	0.607
YOLOv5-5.0	14.4 MB	0.899	0.839	0.829	0.569
YOLOv5-Lite	3.4 MB	0.881	0.824	0.878	0.566
Improved method	28.7 MB	0.936	0.856	0.918	0.585

**Table 8:** Comparison of test results of YOLOv5-6.0, YOLOv5-5.0, YOLOv5-Lite, and improved methods using Pascal VOC2007 public dataset.

Model	Precision	Recall	mAP@0.5	mAP@0.5:0.95
YOLOv5-6.0	0.738	0.622	0.689	0.457
YOLOv5-5.0	0.737	0.61	0.653	0.42
YOLOv5-Lite	0.727	0.628	0.689	0.425
Improved method	0.792	0.595	0.668	0.441

## Data Availability

Not applicable.
